# CARD9 attenuates Aβ pathology and modifies microglial responses in an Alzheimer’s disease mouse model

**DOI:** 10.1073/pnas.2303760120

**Published:** 2023-06-05

**Authors:** Hannah Ennerfelt, Coco Holliday, Daniel A. Shapiro, Kristine E. Zengeler, Ashley C. Bolte, Tyler K. Ulland, John R. Lukens

**Affiliations:** ^a^Department of Neuroscience, Center for Brain Immunology and Glia, University of Virginia, Charlottesville, VA 22908; ^b^Neuroscience Graduate Program, University of Virginia, Charlottesville, VA 22908; ^c^Cell and Molecular Biology Graduate Training Program, University of Virginia, Charlottesville, VA 22908; ^d^Department of Microbiology, Immunology and Cancer Biology, University of Virginia, Charlottesville, VA 22908; ^e^Medical Scientist Training Program, University of Virginia, Charlottesville, VA 22908; ^f^Department of Pathology and Laboratory Medicine, University of Wisconsin, Madison, WI 53705

**Keywords:** Alzheimer’s disease, microglia, CARD9, amyloid beta, neuroimmunology

## Abstract

Recent advances have highlighted the importance of several innate immune receptors expressed by microglia in Alzheimer’s disease (AD). In particular, mounting evidence from AD patients and experimental models indicates pivotal roles for TREM2, CD33, and CD22 in neurodegenerative disease progression. While there is growing interest in targeting these microglial receptors to treat AD, we still lack knowledge of the downstream signaling molecules used by these receptors to orchestrate immune responses in AD. Notably, TREM2, CD33, and CD22 have been described to influence signaling associated with the intracellular adaptor molecule CARD9 to mount downstream immune responses outside of the brain. However, the role of CARD9 in AD remains poorly understood. Here, we show that genetic ablation of CARD9 in the 5xFAD mouse model of AD results in exacerbated amyloid beta (Aβ) deposition, increased neuronal loss, worsened cognitive deficits, and alterations in microglial responses. We further show that pharmacological activation of CARD9 promotes improved clearance of Aβ deposits from the brains of 5xFAD mice. These results help to establish CARD9 as a key intracellular innate immune signaling molecule that regulates Aβ-mediated disease and microglial responses. Moreover, these findings suggest that targeting CARD9 might offer a strategy to improve Aβ clearance in AD.

## Significance

Although significant progress in recent years has been made in defining key innate immune receptors involved in Alzheimer’s disease (AD), our knowledge of the specific intracellular signaling molecules that coordinate immune responses in AD remains poorly defined. In these studies, we have identified a previously undescribed role for the innate immune signaling molecule CARD9 in the 5xFAD mouse model of AD. We specifically demonstrate that CARD9 deletion in 5xFAD mice leads to impaired control of Aβ, worsened cognitive decline, and aberrant microglial activation. We further show that pharmacological activation of CARD9 boosts Aβ clearance in the hippocampus. Collectively, these findings uncover CARD9 as a molecular signaling molecule used by the innate immune system in Aβ-mediated neurological disease.

Alzheimer’s disease (AD) is a neurogenerative disorder characterized by amyloid beta (Aβ) accumulation, the seeding of neurofibrillary tangles, and neuroinflammation (1, 2). The culmination of these pathologies leads to neuronal loss and memory decline in patients (3). Microglia, the resident immune cells of the brain, have been heavily implicated in AD pathogenesis. More specifically, genome-wide association studies (GWAS) have identified that a large percentage of late-onset AD risk loci affect genes involved in microglial biology and function (e.g., *TREM2* and *CD33*) (4). Importantly, recent findings from AD mouse models have further solidified pivotal roles for microglia in Alzheimer’s-related disease pathogenesis (5–11). It is currently thought that microglia affect AD progression through various mechanisms that include cytokine production, phagocytosis of Aβ and other neurotoxic agents, and compaction/containment of Aβ plaques. While these studies clearly define microglia as important players in AD, we are only just beginning to appreciate the molecular and effector mechanisms that microglia employ to influence neurodegenerative disease. For instance, despite our ample understanding of which microglial receptors are involved in modulating AD progression, much of the intracellular signaling employed by microglia in the AD brain environment remains poorly described.

Notably, both human genetics studies and work in AD animal models have begun to uncover pivotal roles for a number of ITAM (immunoreceptor tyrosine-based activation motif)- and ITIM (immunoreceptor tyrosine-based inhibition motif)-containing receptors in AD pathogenesis. For example, emerging evidence indicates critical roles for the ITAM-containing receptor triggering receptor expressed on myeloid cells 2 (TREM2), as well as the ITIM-containing receptors sialic acid-binding immunoglobulin-like lectin 3 (CD33) and sialic acid-binding immunoglobulin-like lectin 2 (CD22) in AD (12–35). In the context of peripheral infection models, engagement of ITAM-containing receptors has been shown to promote the activation of caspase recruitment domain-containing protein 9 (CARD9) signaling and subsequent upregulation of cytokine production (36–38). In contrast, stimulation of ITIM-containing receptors provokes SHP-1 activation, which potently inhibits downstream CARD9 signaling and consequently results in the dampening of cytokine production and phagocytosis (36, 37). While increasing evidence indicates that ITAM/ITIM-containing immune receptors (i.e., TREM2, CD33, and CD22) are critically involved in AD progression, we still lack in-depth knowledge of the intracellular signaling molecules employed by these immune receptors to influence neurodegenerative disease. CARD9 has been shown to control immune responses downstream of TREM2, CD33, and CD22 in other models of disease (36–38), although the involvement of CARD9 in AD and most other neurodegenerative disorders remains to be determined.

To explore a potential role for CARD9 in Alzheimer’s-related disease, we crossed CARD9-deficient mice with 5xFAD mice, a well-described mouse model of Aβ-mediated neurological disease. Here, we find that genetic ablation of *Card9* in 5xFAD mice leads to increased Aβ accumulation in the brain, exacerbated neuronal loss, and worsened memory decline compared with 5xFAD littermate controls. Given that CARD9 is nearly exclusively expressed by microglia in the central nervous system (CNS) (39), we observed that microglia in *Card9*^−/−^ 5xFAD mice display dramatically increased proliferation while they simultaneously exhibit impaired morphological activation in response to Aβ plaques. We further show that treating 5xFAD mice with a potent exogenous trigger of CARD9 signaling leads to improved control of Aβ in the hippocampus. Taken together, these findings indicate that the innate immune signaling molecule CARD9 is a regulator of Aβ-mediated neurological disease and further suggest that targeting CARD9 activation may offer a therapeutic strategy to promote Aβ clearance.

## Results

### CARD9 Signaling Restricts Brain Amyloidosis in 5xFAD Mice.

**Fig. 1.**
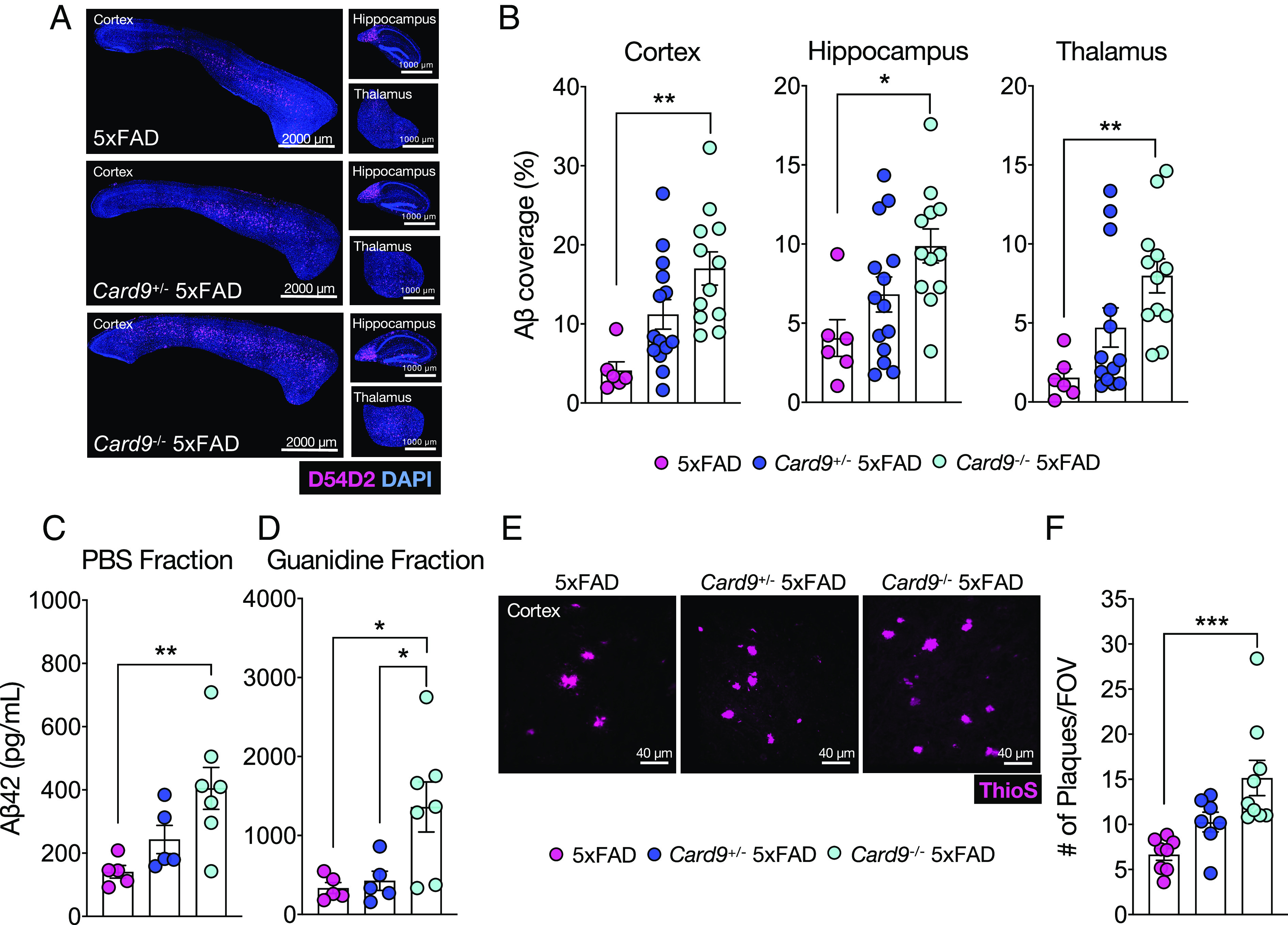
CARD9 deletion leads to increased Aβ accumulation in 5xFAD mice. (*A*–*F*) Brains were harvested from 5-mo-old 5xFAD, *Card9*^+/−^5xFAD, and *Card9*^−/−^5xFAD mice to evaluate Aβ load. (*A*) Representative images of Aβ (D54D2, pink; DAPI, blue) staining from sagittal sections in the cortex, hippocampus, and thalamus. Original magnification: 10x; (scale bar, 2,000 µm and 1,000 µm.) (*B*) Quantification of percent area covered by Aβ in the cortex, hippocampus, and thalamus. Combined data from three independent experiments. (*C* and *D*) Aβ levels detected by Aβ-42 enzyme-linked immunosorbent assay (ELISA). (*C*) Soluble (PBS buffer extraction) and (*D*) insoluble (guanidine extraction) fractions from 5-mo-old 5xFAD, *Card9*^+/−^5xFAD, and *Card9*^−/−^5xFAD half-brain hemispheres. (*E* and *F*) Representative images and quantification of Aβ plaques measuring ThioS^+^ (pink) plaque numbers in the cortex field of view (FOV), with combined data from a total of 50 to 100 plaques from 3 matching brain sections per mouse. Original magnification: 63x; (scale bar, 40 µm.) Statistical significance between experimental groups was calculated by one-way ANOVA with Tukey’s post hoc test (*B*–*D* and *F*). *P < 0.05, **P < 0.01, and ***P < 0.001. Error bars represent mean ± SEM, and each data point represents an individual mouse (*B*–*D* and *F*).

To investigate how CARD9 influences the development of Aβ pathology, we introduced a germline deletion of *Card9* into 5xFAD mice, an AD mouse model characterized by early Aβ accumulation (40, 41). At 5 mo of age, *Card9*^−/−^5xFAD mice had significantly greater Aβ burden in the cortex, hippocampus, and thalamus in comparison to *Card9*^+/−^5xFAD and *Card9*^ +/+^5xFAD (referred to as 5xFAD mice) littermate controls (Fig. 1 *A* and *B*). Consistent with the increased levels of Aβ staining observed in 5xFAD mice that lack CARD9, we detected increased amounts of Aβ42, the most deleterious isoform of Aβ (42, 43), in both the soluble (phosphate buffered saline (PBS)-extracted) and insoluble (guanidine-extracted) brain fractions obtained from *Card9*^−/−^5xFAD mice (Fig. 1 *C* and *D*). The soluble fraction of Aβ is thought to contain the most neurotoxic oligomers of Aβ (44), whereas the insoluble fraction consists of higher order Aβ forms found in amyloid plaques (45). In alignment with the increase of Aβ in the insoluble fraction, the cortex in *Card9*^−/−^5xFAD also contains approximately double the number of individual Aβ plaques in comparison to 5xFAD littermate control mice (Fig. 1 *E* and *F*). These data suggest that CARD9 plays an important role in limiting Aβ accumulation in 5xFAD mice.

### The Loss of CARD9 Exacerbates Neuronal Loss and Memory Decline in 5xFAD Mice.

**Fig. 2.**
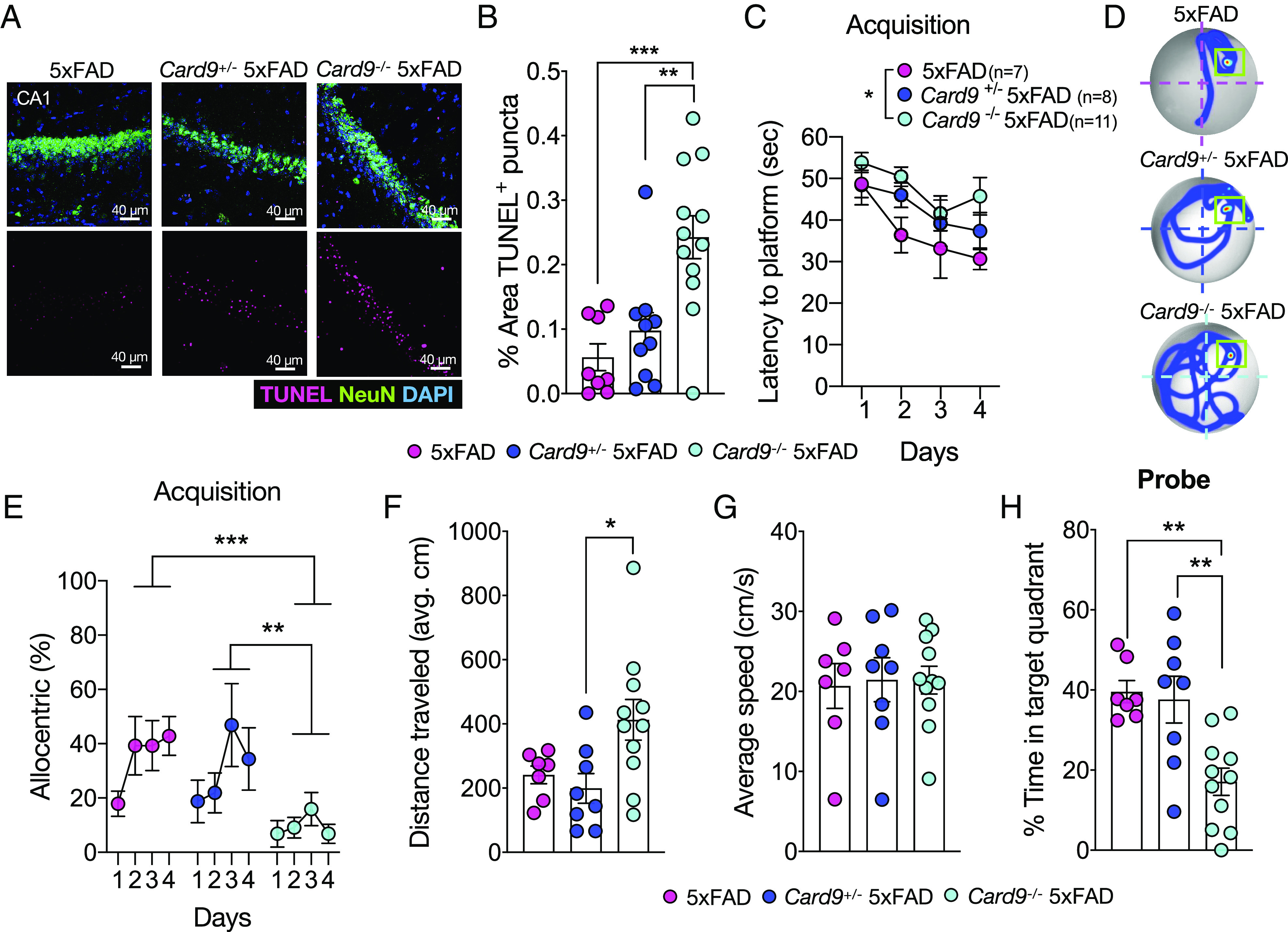
*Card9* deficiency leads to worsened neuronal health and cognitive impairment in 5xFAD mice. (*A* and *B*) Brains were harvested from 5-mo-old 5xFAD, *Card9*^+/−^5xFAD, and *Card9*^−/−^5xFAD mice to assess neuronal death. The CA1 region of the hippocampus was evaluated for neuronal cell death by TUNEL assay (pink), NeuN staining (green), and DAPI (blue). Original magnification: 63x; (scale bars, 40 µm.) (*C*–*H*) The Morris water maze (MWM) test was used to assess spatial learning and memory in 4-mo-old 5xFAD, *Card9*^+/−^ 5xFAD, and *Card9*^−/−^ 5xFAD mice. (*C*–*G*) Acquisition stage of learning in the MWM. (*C*) Latency to platform (acquisition). (*D*) Representative heatmaps of mouse trajectory on day 4 of acquisition with a green box outlining the location of the platform and (*E*) plotted percentage of allocentric navigation strategy during MWM acquisition. (*F*) Distance traveled averaged from all four trials in maze (cm) and (*G*) average speed of travel (cm/s) on day 4 of acquisition. (*H*) Percentage of time spent in the target quadrant (probe). Statistical significance between experimental groups was calculated by repeated-measures two-way ANOVA with Bonferroni’s post hoc test (*C* and *E*) or one-way ANOVA with Tukey’s post hoc test (*B* and *F*–*H*) from three independent experiments. *P < 0.05, **P < 0.01, and ***P < 0.001. Error bars represent mean ± SEM (*B*, *C*, *E*–*H*) and each data point represents an individual mouse (*B* and *F*–*H*), or the average of experimental mice per group (*C* and *E*).

Continual accumulation of Aβ can cause appreciable neuronal damage in the AD brain (2, 46, 47) and the deleterious effects of Aβ deposition on neuronal health is thought to drive behavioral changes and pronounced learning and memory decline (48–51). To determine if the increase in Aβ accumulation seen in *Card9*^−/−^5xFAD mice is accompanied by heightened neuronal loss, we first evaluated neuronal cell death by performing terminal deoxynucleotidyl transferase dUTP nick end labeling (TUNEL) staining on the CA1 region of hippocampal samples from 5xFAD, *Card9*^+/−^5xFAD, and *Card9*^−/−^5xFAD mice. The CA1 region of the hippocampus is densely packed with neurons forming circuits responsible for the consolidation and retrieval of memory (52). Initial seeding of Aβ often originates in the hippocampus; therefore, neuronal damage is often seen in this region, and this is thought to explain some of the learning and memory behavioral deficits commonly observed in Aβ-driven mouse models of AD (53–55). Although 5xFAD mice do not characteristically display marked neuronal loss until later stages of disease (56), 5-mo-old *Card9*^−/−^5xFAD mice were found to have pronounced levels of TUNEL^+^ NeuN^+^ cells in the CA1, indicative of neuronal death (Fig. 2 *A* and *B*). Taken together, these data suggest that impaired control of Aβ in CARD9-deficient 5xFAD mice is associated with increased levels of neuronal cell death in the CA1 region of the hippocampus.

Given the increased neuronal loss and amyloidosis observed in CARD9-deficient 5xFAD mice, we next sought to determine whether *Card9*^−/−^5xFAD mice also display deficits in learning and memory. To this end, we employed the Morris water maze (MWM) behavioral test to probe spatial learning and memory (57). Over the first 4 d of MWM, 5xFAD control mice exhibited a substantial decrease in latency to find the hidden platform which is indicative of intact spatial learning (Fig. 2*C*). In comparison, *Card9*^−/−^5xFAD mice spent more time searching for the hidden platform over the four acquisition days, demonstrating significantly impaired spatial learning in comparison to 5xFAD littermate controls (Fig. 2*C*). The navigation path used by mice to find the platform can also provide insights into the degree of cognitive dysfunction. Allocentric movement, for example, is defined by the ability of the mouse to find the hidden platform within 3 navigational turns and is dependent on hippocampal spatial memory (58). Interestingly, *Card9*^−/−^5xFAD mice displayed significantly decreased hippocampal-based allocentric spatial memory when searching for the hidden platform compared with 5xFAD and *Card9*^+/−^5xFAD littermate control mice (Fig. 2 *D* and *E*). Moreover, *Card9*^−/−^5xFAD mice also covered a significantly longer distance in search of the hidden platform on the fourth day of the MWM test (Fig. 2*F*), suggesting a less targeted platform search in comparison to 5xFAD controls. Locomotor deficits did not contribute to any differences seen between groups in the MWM acquisition days, as comparable speeds were measured between 5xFAD, *Card9*^+/−^5xFAD, and *Card9*^−/−^5xFAD mice (Fig. 2*G*). Impaired spatial memory was also displayed by *Card9*^−/−^5xFAD mice during probe day, as they spent significantly less time than 5xFAD and *Card9*^+/−^5xFAD mice in the MWM pool target quadrant that had contained the hidden platform during the first 4 d of acquisition (Fig. 2*H*). Importantly, CARD9 deletion alone in the absence of Aβ amyloidosis was not found to appreciably impact performance in the MWM test and we observed comparable spatial learning and memory in *Card9*^+/+^, *Card9*^+/−^, and *Card9*^ −/−^ mice (*SI Appendix*, Fig. S1). These findings suggest that absence of CARD9 in 5xFAD mice leads to increased levels of neuronal cell death in the hippocampus and accelerated cognitive impairments.

### CARD9 Deficiency in 5xFAD Mice Results in Altered Microglial Responses.

In recent years, there has been ever-growing interest in the roles that microglia play in AD. This was initially sparked by results obtained in human genetics studies linking mutations in multiple microglial genes to late-onset AD (4). Follow-up studies in AD mouse models have largely confirmed that microglia can influence various aspects of AD-related pathology (14, 21, 22, 32, 35). Notably, mounting evidence suggests that microglia are critically involved in both the phagocytosis and compaction of Aβ, and that this subsequently helps to protect neurons from interacting with neurotoxic species of Aβ (30, 59). Therefore, given our findings demonstrating increased levels of Aβ and neuronal loss in CARD9-deficient 5xFAD mice, paired with the knowledge that *Card9* is primarily expressed by microglia in the brain, we were interested in how CARD9 deletion affects the mobilization of microglial responses to Aβ-driven pathology.

The observed increase in Aβ load in the *Card9*^−/−^5xFAD brain prompted us to investigate potential differences in the phagocytic capacity of macrophages in *Card9*-deficient mice. To begin, we generated bone marrow-derived macrophages (BMDMs) from *Card9*^+/+^ (referred to as WT) and *Card9*^−/−^ mice. WT and *Card9*^−/−^ BMDMs were stimulated with oligomeric Aβ tagged with CyPher5E, a pH sensitive dye. CypHer5E fluoresces when brought into the low-pH environment of the phagolysosome; consequently, an increased staining of CypHer5E suggests elevated phagocytosis of Aβ by BMDMs. In these studies, we found that CARD9 deletion did not appreciably impact Aβ uptake by BMDMs (*SI Appendix*, Fig. S2). To discern whether *Card9*-deficiency affects Aβ clearance in vivo, we next injected CypHer5E-tagged Aβ into the cortex of WT and *Card9*^−/−^ mice and compared the presence of CypHer5E relative to total Aβ in the injection site. Similar to our in vitro macrophage findings, we did not observe substantial differences in the clearance of Aβ by IBA1^+^ cells between WT and *Card9*^−/−^ mice at 48 h postintracortical injection of CypHer5E-tagged Aβ (*SI Appendix*, Fig. S2). Collectively, these results suggest that *Card9*-deficiency does not appreciably affect the phagocytosis of Aβ by BMDMs or CNS-resident cells.

**Fig. 3.**
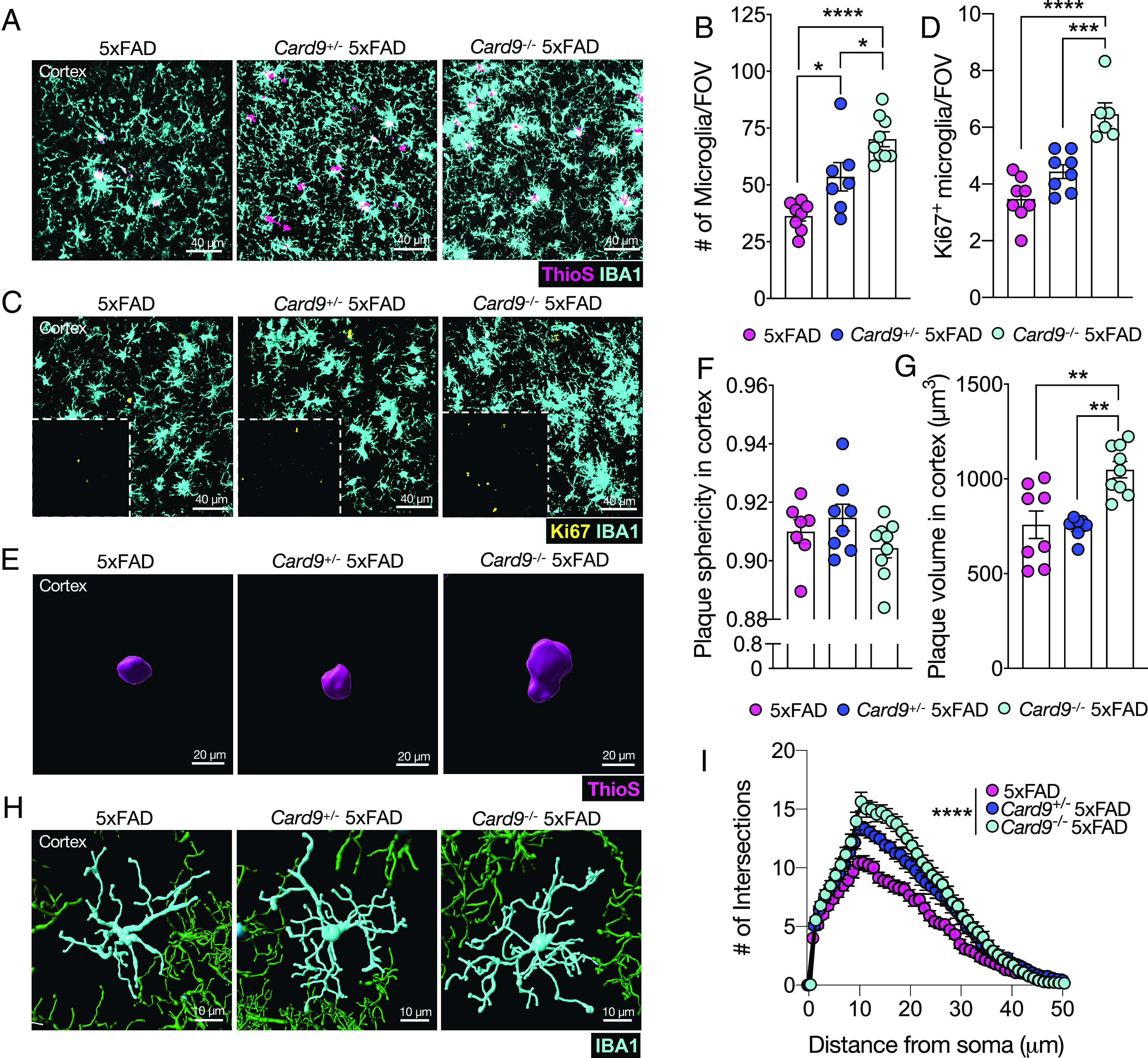
The loss of *Card9* leads to altered microgliosis in 5xFAD mice. (*A*–*I*) Brains were harvested from 5-mo-old 5xFAD, *Card9*^+/−^5xFAD, and *Card9*^−/−^5xFAD mice to assess microglial activity and Aβ plaque volume. (*A* and *B*) Representative images and quantification of microglia numbers (IBA1, cyan) surrounding ThioS^+^ plaques (pink) in the field of view (FOV) of the frontal cortex of 5xFAD, *Card9*^+/−^5xFAD, and *Card9*^−/−^5xFAD mice. Original magnification: 63x; (scale bar, 40 µm.) (*C* and *D*) Representative images of microglial proliferation measured by evaluating Ki67 (yellow) colocalization with IBA1^+^ (cyan) microglia in the cortex of matched sagittal sections. Original magnification: 63x; (scale bar, 40 µm.) (*E*–*G*) Representative images and quantification of ThioS-labeled (pink) plaque sphericity and volume in the cortex. Original magnification: 63x; (scale bar, 20 µm.) (*F*) Quantification of plaque sphericity with 1.00 being the most spherical. (*G*) Quantification of plaque volume in the cortex. (*H* and *I*) Microglial morphology calculated by Sholl analysis from a total of 12 microglia from 3 matching brain sections per mouse (n = 5 mice per group). (*H*) Representative microglia renderings and (*I*) Sholl analysis. Original magnification: 63x; (scale bar, 10 µm.) Statistical significance between experimental groups was calculated by one-way ANOVA with Tukey’s post hoc test (*B*–*D*, *F*, and *G*) or repeated-measures two-way ANOVA with Bonferroni’s post hoc test (*I*). *P < 0.05, **P < 0.01, ***P < 0.001, ****P < 0.0001. Error bars represent mean ± SEM (*B*–*D*, *F*, *G*, and *I*). Each data point represents an individual mouse (*B*–*D*, *F*, and *G*) or the average of 5 mice (*I*). Data were collected from 6 fields of view (FOV) from a total of 3 matched sagittal sections (*B* and *D*) or 50 to 100 plaques from the cortex of each mouse (*F* and *G*).

To our surprise, we found that the loss of *Card9* results in a step-wise increase in the number of IBA1-labeled microglia within the cortex of 5xFAD mice (Fig. 3 *A* and *B*). This progressive increase in microglia between 5xFAD, *Card9*^+/−^5xFAD, and *Card9*^−/−^5xFAD mice is suggestive of a gene-dosage effect for the *Card9* allele (Fig. 3 *A* and *B*). The approximate doubling of microglial numbers in the cortex of *Card9*^−/−^5xFAD mice compared with 5xFAD controls is likely explained by a similar magnitude increase in the number of proliferating microglia (Fig. 3 *C* and *D*). More specifically, *Card9*^−/−^5xFAD mice have approximately twice the number of Ki67^+^ microglia in their cortex compared with 5xFAD mice (Fig. 3 *C* and *D*). In contrast, we did not observe any appreciable differences in microglial numbers or Ki67 expression between CARD9-deficient and wild-type mice that lacked the 5xFAD transgenes (*SI Appendix*, Fig. S3), suggesting that the increased numbers of microglia seen in *Card9*^−/−^5xFAD mice was likely driven by Aβ-associated pathology.

The stimulation of microglial recruitment and proliferation in response to AD pathology is well characterized (22, 60), however, the loss of CARD9 appears to significantly enhance microglial mobilization to Aβ plaques while concurrently exacerbating Aβ load in the cortex of 5xFAD mice. One potential explanation for these somewhat paradoxical findings of increased numbers of both microglia and Aβ load in *Card9*^−/−^5xFAD mice is that the microglia recruited to Aβ in CARD9-deficient 5xFAD mice are less effective in influencing Aβ compaction. To test this, we evaluated both Aβ plaque volume and sphericity as a readout of the ability of CARD9-sufficient and -deficient microglia to shape Aβ plaque compaction (11, 22, 59). For instance, more compact plaques are believed to indicate the formation of a functional microglial barrier in which microglia physically interact with the plaque to decrease the Aβ footprint in the brain parenchyma (22, 61). While we did not observe any notable differences in Aβ plaque sphericity between 5xFAD and *Card9*^−/−^5xFAD mice (Fig. 3 *E* and *F*), we did, however, observe that CARD9 deficiency in 5xFAD mice leads to increased plaque volume when compared to 5xFAD littermate controls (Fig. 3 *E* and *G*).Therefore, despite the increase in microglia numbers in *Card9*^−/−^5xFAD mice, *Card9*-deficient microglia failed to reduce the extent of Aβ plaque volume, suggesting that CARD9 is critical for microglial containment of Aβ.

It has been extensively shown that microglia become less ramified and more ameboid in morphology in response to Aβ, and this is thought to affect the ability of microglia to modulate Aβ load (62). Because *Card9*^−/−^5xFAD microglia exhibit increased proliferation without curbing Aβ load, we were also interested in investigating whether *Card9*-deficiency impacts the ability of microglia to undergo morphological changes in response to Aβ-associated pathology. To explore this in greater detail, we focused on the Aβ-rich cortex and identified progressive microglial morphological differences between 5xFAD, *Card9*^+/−^5xFAD, and *Card9*^−/−^5xFAD mice (Fig. 3 *H* and *I*). For instance, *Card9*^+/−^5xFAD and *Card9*^−/−^5xFAD microglia displayed significantly more complex morphology as measured by Sholl analysis compared with 5xFAD microglia (Fig. 3 *H* and *I*). In contrast, *Card9*^+/+^, *Card9*^+/−^, and *Card9*^−/−^ microglia did not exhibit morphological changes in the absence of Aβ (*SI Appendix*, Fig. S3). Therefore, CARD9 is critical in driving microglial morphological activation upon Aβ stimulation. Collectively our findings suggest that CARD9 regulates microglial proliferation and morphological activation as well as Aβ compaction in the 5xFAD brain.

### Microglial ROS Production and Lipid Droplet Formation Are Not Impacted by CARD9 Deletion in 5xFAD Mice.

While microglia can play beneficial roles in AD-related disease through their critical involvement in Aβ containment and disposal, unchecked activation of microglial inflammatory responses can also have deleterious effects and perpetuate further AD pathogenesis (8, 46). To gain a deeper understanding of how CARD9 may impact microglial-induced inflammation, we began by investigating whether *Card9*^−/−^5xFAD microglia take on lipid-droplet-accumulating microglia (LDAM) properties. LDAMs have been previously described in the aged brain as pro-inflammatory, with increased reactive oxygen species (ROS) and cytokine production (63). To determine if CARD9-deficient microglia in the 5xFAD brain exhibit LDAM-related dysregulation, we harvested brains from 5xFAD and *Card9*^−/−^5xFAD mice and measured levels of BODIPY, a dye that labels lipid-droplets, and CellROX, a dye that fluoresces when oxidized by ROS, in CD11b^hi^CD45^int^ cells. However, flow cytometric analysis revealed no appreciable differences in BODIPY or CellROX staining in *Card9*^−/−^5xFAD microglia when compared to 5xFAD control microglia (*SI Appendix*, Fig. S4). Thus, *Card9*-deficiency does not significantly increase the prevalence of LDAMs or ROS production by microglia in the 5xFAD brain.

Aberrant production of pro-inflammatory cytokine production by microglia is also believed to contribute to the propagation of AD pathology and memory deficits. For instance, the cytokines IL-6, IL-1β, and IL-18 are often elevated in AD and have been reported to provoke Aβ accumulation and cognitive decline (64–67). CARD9 is known to regulate NF-κB activation, a transcription factor that coordinates the production of several pro-inflammatory cytokines including IL-6, pro-IL-1β, and pro-IL-18 (68). Therefore, we investigated the levels of key pro-inflammatory and anti-inflammatory cytokines in the whole brain of 5xFAD, *Card9*^+/−^5xFAD, and *Card9*^−/−^5xFAD mice. However, we did not observe altered levels of IL-1α, IL-1β, IL-4, IL-6, IL-10, IL-17, IFN-γ, and TNF-α by multiplex ELISA (*SI Appendix*, Fig. S4). Altogether, these findings suggest that CARD9 deletion in 5xFAD mice does not substantially influence pro-inflammatory cytokine levels, production of ROS, or lipid droplet accumulation by microglia.

### Effects of CARD9 Deletion on the Microglial Transcriptional Response in 5xFAD Mice.

**Fig. 4.**
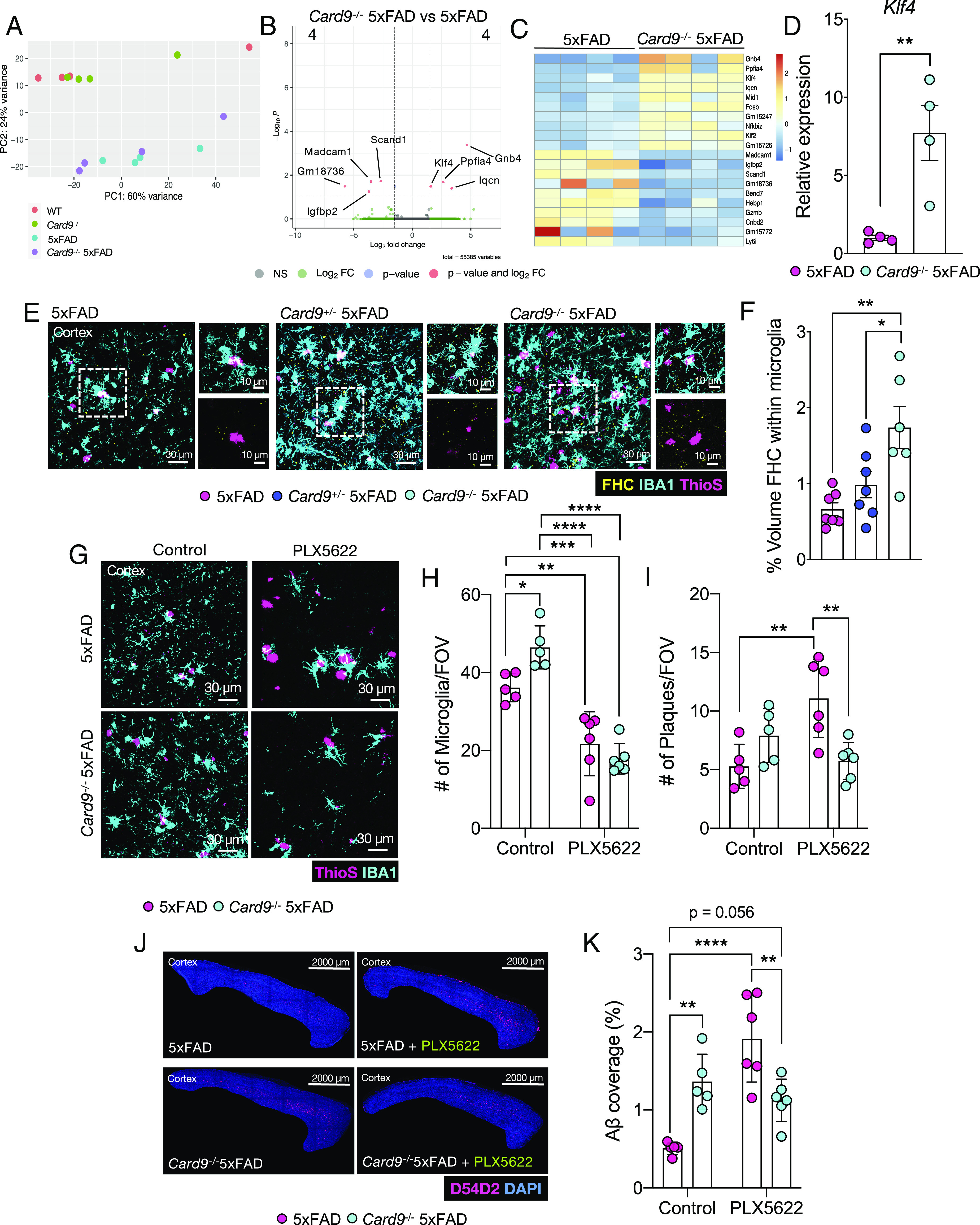
*Card9*-dependent microglial regulation in 5xFAD mice. (*A*–*C*) RNA-Seq was performed on microglia from 5-mo-old *Card9^+^*^/+^ (denoted as WT), *Card9*^−/−^, 5xFAD, and *Card9*^−/−^5xFAD mice sorted from single-cell brain suspensions using anti-CD11b^+^-coated magnetic beads and magnetic column sorting. (*A*) Principal component (PC) analysis of sample clustering. (*B*) Volcano plot comparing differentially expressed genes (FDR < 0.1) between *Card9*^−/−^5xFAD and 5xFAD microglia, where 4 genes are significantly down-regulated and 4 genes are significantly up-regulated. (*C*) Heatmap representation of the top 10 overall up-regulated and down-regulated genes between microglia isolated from *Card9*^−/−^5xFAD and 5xFAD mice. (*D*) qPCR validation of *Klf4* expression in microglia sorted from *Card9*^−/−^5xFAD and 5xFAD mice. (*E* and *F*) Brains were harvested from 5-mo-old 5xFAD, *Card9*^+/−^5xFAD, and *Card9*^−/−^5xFAD mice to assess microglial ferritin heavy chain (FHC) levels in the cortex. (*E*) Representative images and (*F*) quantification of FHC (yellow) within microglia (IBA1, cyan) surrounding ThioS^+^ plaques (pink) in the field of view (FOV) of the frontal cortex of 5xFAD, *Card9*^+/−^5xFAD, and *Card9*^−/−^5xFAD mice. Original magnification: 63x; (scale bar, 30 µm and 10 µm.) (*G*–*K*) 5xFAD and *Card9*^−/−^5xFAD mice were fed normal chow (denoted as control) or PLX5622 chow beginning at 1.5 mo of age and harvested at 4 mo of age. (*G*) Representative images and (*H*) quantification of microglia numbers (IBA1, cyan) surrounding ThioS^+^ plaques (pink) in the FOV of the frontal cortex of normal chow- and PLX5622 chow-fed 5xFAD and *Card9*^−/−^5xFAD mice. (*G*) Representative images and (*I*) quantification of ThioS^+^ Aβ plaque (pink) numbers in the cortex FOV, with combined data from a total of 30 to 75 plaques from 3 matching brain sections per mouse. Original magnification: 63×; (scale bar = 30 µm.) (*J*–*K*) Representative images of Aβ (D54D2, pink; DAPI, blue) staining from sagittal sections in the cortex of normal chow- and PLX5622 chow-fed 5xFAD and *Card9*^−/−^5xFAD mice. Original magnification: 10×; (scale bar, 2,000 µm.) Statistical significance between experimental groups was calculated by unpaired two-tailed Student’s *t* test (*D*), one-way ANOVA with Tukey’s post hoc test (*F*), and two-way ANOVA with Tukey’s post hoc test (*H*, *I*, and *K*). *P < 0.05, **P < 0.01, ***P < 0.001, and ****P < 0.0001. Error bars represent mean ± SEM (*D*, *F*, *H*, *I*, and *K*). Each data point represents an individual mouse (*D*, *F*, *H*, *I*, and *K*).

Microglia exposed to AD-associated pathology are thought to undergo a transcriptional shift to become disease-associated microglia (DAMs) (69). Upon stimulation, DAMs down-regulate their homeostatic markers and subsequently up-regulate several activation markers in a biphasic manner (70). This process of DAM acquisition is thought to supply microglia with an increased capacity to respond to and eliminate AD pathology (71). Given that *Card9*-deficiency exacerbates disease progression in 5xFAD mice, we hypothesized that the microglia of *Card9*^−/−^5xFAD mice would be unable to undergo the transcriptional evolution seen in DAMs. Thus, to evaluate how CARD9 may affect microglia activity in an unbiased and comprehensive manner, we performed bulk RNA sequencing (RNA-Seq) on magnetic bead-sorted CD11b^+^ cells from the brains of *Card9*^+/+^ (WT), *Card9*^−/−^, 5xFAD, and *Card9*^−/−^5xFAD mice. Principal component (PC) analysis uncovered a prominent separation between homeostatic (WT and *Card9*^−/−^) and 5xFAD (5xFAD and *Card9*^−/−^5xFAD) microglia (Fig. 4*A*).

To our surprise, we did not observe a major effect of CARD9 deletion on the microglial transcriptional response in 5xFAD mice (Fig. 4 *B* and *C*). In fact, when comparing *Card9*^−/−^5xFAD microglia to 5xFAD microglia, we only detected 4 down-regulated (*Madcam1*, *Igfbp2*, *Scand1*, and *Gm18736*) and 4 up-regulated (*Gnb4*, *Ppfia4, Klf4*, and *Iqcn*) genes (false discovery rate (FDR) < 0.1) (Fig. 4 *B* and *C*). However, we found the upregulation of *Klf4* in *Card9*^−/−^5xFAD microglia analyzed by RNA-Seq and qPCR (Fig. 4 *C* and *D*) compelling due to the fact that KLF4 has been shown to regulate microglia-driven neuroinflammation and neuronal loss in the context of AD pathology (72, 73). Although we did not observe overt changes in inflammatory signaling or oxidative stress between 5xFAD and *Card9*^−/−^5xFAD mice (Fig. 4 and *SI Appendix*, Fig. S4) which have previously been described to be regulated by KLF4, we did observe a significant increase in neuronal cell death in the CA1 region of the hippocampus (Fig. 2 *A* and *B*). Neuronal cell loss is a key element contributing to AD progression that is reported to be propagated by KLF4 (72–75). Interestingly, KLF4 is believed to contribute to microglial iron dyshomeostasis that promotes impaired microglial response to Aβ (73). In fact, iron-laden microglia have been identified in the AD brain (76). Therefore, we looked at ferritin heavy chain accumulation in microglia, a subunit of ferritin critical for iron sequestration (77). We took into account the increase in microglial coverage in the cortex of *Card9*^−/−^5xFAD mice by normalizing the volume of ferritin heavy chain to the total volume of IBA1^+^ cells; thus, allowing us to calculate ferritin heavy chain level per microglia. Interestingly, we observed a striking increase in ferritin heavy chain staining within *Card9*^−/−^5xFAD microglia compared with 5xFAD controls (Fig. 4 *E* and *F*). Thus, CARD9 likely regulates iron homeostasis in 5xFAD microglia. It is important to note that *Card9*-deletion at steady state in the absence of 5xFAD transgene did not cause a major transcriptional shift in microglia, with a unique 4 down-regulated (*Ly6i*, *Hebp1, Tppp3*, and *Cd300e*) and 4 up-regulated (*Cacnb2*, *Prkcq*, *Thnsl1*, and *Ppfia4*) genes compared with WT microglia (FDR < 0.1) (*SI Appendix*, Fig. S5). These findings indicate that CARD9 deletion in 5xFAD mice leads to elevated expression of KLF4 in the brain and that this is associated with increased production of ferritin heavy chain by IBA1+ cells.

### The Impact of Card9 Deletion on Aβ Load Does Not Rely on Increased Numbers of Microglia.

Microglia are known to play both beneficial and detrimental roles in the context of Aβ pathology (5, 6, 21, 69, 78, 79). For instance, microglia are able to shield neurons from neurotoxic species of Aβ (11, 30); however, recent studies also suggest that microglia can contribute to the pathogenic spread of Aβ amyloidosis (6, 80). Therefore, the possibility exists that the increased microgliosis observed in *Card9*-deficient 5xFAD mice (Fig. 3 *A* and *B*) may contribute to the exacerbated AD pathology progression seen in *Card9*^−/−^5xFAD mice (Fig. 1).

To test this, we fed 1.5-mo-old 5xFAD and *Card9*^−/−^5xFAD mice with either normal chow or food containing the CSF1R inhibitor PLX5622 to deplete microglia. Mice remained on PLX5622 chow or normal feed for 2.5 mo and at 4 mo of age the brains were collected to evaluate microgliosis and Aβ load. Importantly, we observed similar levels of microglia depletion in 5xFAD and *Card9*^−/−^5xFAD mice after 2.5 mo of continuous treatment with PLX5622 food (Fig. 4 *G* and *H*). PLX5622-treated 5xFAD mice were found to have significantly increased levels of Aβ plaque load in the cortex when compared with 5xFAD mice on normal chow (Fig. 4 *G*, *I*, *J*, and *K*). In contrast, microglia depletion in *Card9*^−/−^5xFAD mice did not significantly impact Aβ plaque load, as the coverage of Aβ in the cortex was commensurate between PLX5622-treated and control chow-treated *Card9*^−/−^5xFAD mice (Fig. 4 *G*, *I*, *J*, and *K*). These collective findings suggest that the increased numbers of microglia are likely not causative in the exacerbated AD pathology development of CARD9-deficient mice.

### The Ability of β-glucan Treatment to Reduce Aβ Load in 5xFAD Mice Is Dependent on CARD9.

Thus far, we have demonstrated that CARD9 deletion is detrimental for microglial activation and pathology progression in the 5xFAD mouse model. These findings suggest that CARD9 activity plays a protective role in response to Aβ pathology; thus, we sought to explore whether CARD9 stimulation can reduce Aβ load in the brain. We chose to enhance CARD9 signaling by targeting the activation of CLEC7A, a receptor that has been extensively reported to promote CARD9 activation in response to fungal triggers (81, 82). CLEC7A can initiate downstream CARD9 signaling following its binding to β-D-glucans, a component of yeast cell walls (83). We injected pustulan, a β-D-glucan that can activate a range of fungal recognition signaling pathways including CARD9, into the Aβ-laden hippocampus of 5xFAD mice to evaluate how the activation of the CLEC7A-CARD9 pathway affected Aβ load.

**Fig. 5.**
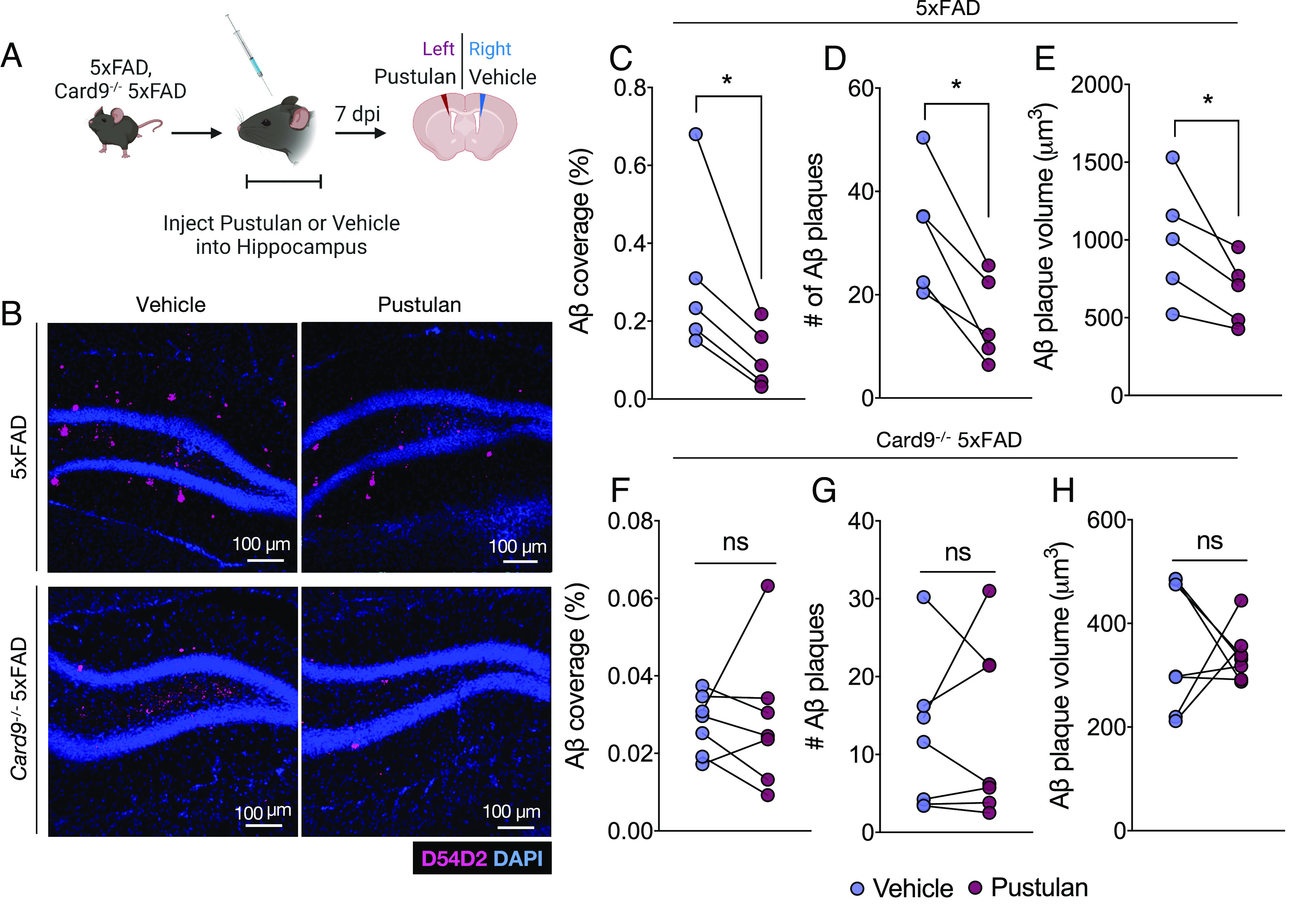
Pustulan treatment enhances Aβ clearance in a CARD9-dependent manner in the hippocampus of 5xFAD mice. (*A*–*I*) *Right* and *Left* hippocampal injection of vehicle or pustulan, respectively, into 2-mo-old 5xFAD and *Card9*^−/−^ 5xFAD mice. Brains were then harvested 7 days post injection (dpi). (*A*) Experimental design schematic. (*B*) Representative images of Aβ (D54D2, pink) coverage in 2-mo-old 5xFAD and *Card9*^−/−^ 5xFAD mice 7 d following intrahippocampal injection of vehicle or 2 μg pustulan. (*C*–*H*) Quantification of Aβ in the hippocampus of (*C*–*E*) 5xFAD and (*F*–*H*) *Card9*^−/−^ 5xFAD mice. Statistical significance between experimental groups was calculated by paired Student’s *t* test (*C*–*H*). ns = nonsignificant, *P < 0.05. Representative data from 2 independent experiments (*C*–*H*). Error bars represent mean ± SEM, and each data point represents an individual mouse (*C*–*H*).

Two-month-old 5xFAD and *Card9*^−/−^5xFAD mice were injected with either vehicle or 2 μg pustulan into the right and left hemisphere of the hippocampus, respectively. *Card9*^−/−^5xFAD mice were included in these studies as controls to test for any potential off-target and CARD9-independent effects of pustulan treatment on Aβ pathology. Brains were then harvested at 7 d post injection, and Aβ plaque load was compared between the vehicle- and pustulan-treated hemispheres of the hippocampus (Fig. 5*A*). We chose to treat mice at 2 mo of age due to the appreciable Aβ deposition in the hippocampus of mice at this time point. Moreover, there are not yet statistically significant differences in Aβ load between 5xFAD mice and *Card9*^−/−^5xFAD mice at this age, which enables us to more accurately assess whether pustulan has any CARD9-independent effects. In 5xFAD mice, the hippocampal hemisphere treated with pustulan had significantly reduced Aβ coverage, fewer plaques, and lower plaque volume compared to the vehicle-treated hippocampal hemisphere (Fig. 5 *B*–*E*). In contrast, the hippocampi of *Card9*^−/−^5xFAD mice had comparable levels of Aβ regardless of treatment (Fig. 5 *B* and *F*–*H*), suggesting that pustulan promotes Aβ control in a CARD9-dependent manner. Taken together, these data indicate that CARD9 signaling contributes to the restriction of Aβ-driven pathology in the 5xFAD mouse model of AD.

## Discussion

The identification of microglial AD risk genes from GWAS studies has implicated several receptors in both aberrant and protective microglial responses (4, 70, 84). The characterization of these microglial receptors in mice and humans has uncovered several potential therapeutic targets (85–87); the activation or inhibition of these receptors has dominated microglia-targeted AD therapy as they extensively influence microglial response to AD pathology (6, 14, 20, 22, 32, 35, 59). In particular, activation of the TREM2 receptor has been demonstrated to exert neuroprotective effects and has advanced to phase 2 clinical trials for AD treatment (85). However, much of the microglial downstream signaling in AD remains poorly defined, potentially leaving a stone unturned for additional and more precise interventions. The intricacies of microglial function in AD, and how best to target them, are more likely to be uncovered by elucidating the unique and shared downstream molecular underpinnings of these receptors.

In our studies presented here, we identify CARD9, an immune molecule downstream of several microglial receptors implicated in AD, as an important regulator of microglial activation in the context of Aβ-driven pathology in 5xFAD mice. We demonstrate that CARD9 regulates the microglial response to Aβ in the 5xFAD brain, ultimately impacting Aβ load and neuronal loss. In addition, we find that CARD9 protects against cognitive impairment in 5xFAD mice. A transcriptional comparison between 5xFAD and *Card9*^−/−^5xFAD microglia uncovered increased expression of *Klf4* in CARD9-deficient microglia, a transcription factor linked with increased neuronal loss and iron dyshomeostasis (72, 73, 75). Interestingly, in addition to increased neuronal loss seen in the hippocampus, CARD9-deficient 5xFAD mice also display significantly higher iron retention in their microglia, a phenotype emblematic of microglial dysfunction (88, 89). Furthermore, we demonstrate that pustulan-induced activation of CARD9 drives a reduction in Aβ plaque load in the hippocampus of 5xFAD mice. Together, our studies suggest an important role for CARD9 in regulating microglial activation and Aβ load in 5xFAD mice.

CARD9 has classically been defined for its roles in driving myeloid cell inflammatory responses in the context of peripheral fungal infection (68, 83, 90, 91). The ability of microglia to function in the context of infection has long been known (92–95), but whether this mirrors microglial responses to AD pathology remains ill-defined. Interestingly, artifacts of fungal pathogens adjacent to Aβ plaques have been found in the brains of AD patients (96, 97), and although it remains controversial whether fungal infection precedes or follows AD onset (98), microglia are well equipped to recognize and respond to this pathogen in the brain (92). Thus, microglia contain sophisticated machinery that responds to both infectious triggers and AD pathology (8, 10, 94, 99); in turn, microglia have the potential to act as a critical bridge between immune activation and AD pathogenesis. For example, CLEC7A, a receptor upstream of CARD9, is up-regulated by microglia surrounding fungal aggregates and Aβ plaques (35, 99). Interestingly, fungal pathogens contain an amyloid-like structure on their cell surface that allows for pathogen adhesion and biofilm formation (100, 101), which may explain the multimodal upregulation of a fungal receptor such as CLEC7A. Altogether, the characterization of this innate immune response in both modalities of brain infection and neurodegeneration may uncover enigmatic etiologies of AD.

Targeting microglia in AD has become increasingly more complex as scientists gain a more complete understanding of the pleiotropic roles that microglia play in the evolution of disease. Just as AD can be stimulated by vastly heterogeneous triggers involving a combination of aging, environmental factors, and genetics (84, 102–105), microglial functions can also be significantly affected by these same contributing factors. Importantly, the progression of microglial transcription throughout AD pathogenesis has the capacity to significantly alter microglial responses and subsequent AD progression (33, 35, 69, 70, 106). Recent work has described the importance of these innate myeloid cells taking on a DAM signature during AD, promoting enhanced phagocytic and inflammatory function (69). However, more recent work has demonstrated that microglia undergo a phasic shift from phagocytic and proliferative to antiviral and apoptotic in the context of tauopathy (106). In the latter phase, microglia are immunosuppressed, but also contribute to chronic inflammation, a pathogenic hallmark in the AD brain (106). Ultimately, effectively targeting microglia in AD, either through the enhancement or diminution of microglial function, may largely depend on the prevailing pathology, stage of disease, and the delineation of influential microglial signaling.

Altogether, differential microglial responses in AD, including proliferation, activation, and pathology clearance, involve critical downstream signaling that have remained poorly described to date. In the studies presented here, we have demonstrated that CARD9 functions to impede AD pathology progression and acts as a relevant intracellular mediator of microglial response to Aβ in 5xFAD mice. Our findings highlight the important nature of CARD9, a shared downstream molecule of several AD-associated microglial receptors, in protecting against Aβ-mediated neurodegeneration.

## Materials and Methods

### Mice.

All mouse experiments were conducted in accordance with the relevant regulations and guidelines of the University of Virginia (UVA) and approved by the UVA Animal Care and Use Committee. Female 5xFAD mice (Stock # 34848-JAX)*, Card9^−/−^* mice (Stock # 028652), and C57BL/6J mice (Stock # 000664) were obtained from The Jackson Laboratory and were crossed to generate *Card^+/+^* (denoted as WT), *Card^+/−^*, *Card9^−/−^*, *Card^+/+^*5xFAD (denoted as 5xFAD), *Card^+/−^*5xFAD, and *Card9^−/−^*5xFAD experimental mice. Mice underwent a 12-h light/dark cycle, were housed in a specific pathogen-free vivarium and at a standardized humidity (50 ± 10%) and temperature (21 ± 1.5 °C). For CSF1R inhibitor experiments, mice were given PLX5622 diet (Research Diets Inc., D19101002i) for 2.5 mo.

### Brain Tissue Harvest.

Experimental mice underwent euthanasia approved by the University of Virginia Animal Care and Use Committee. Mice were euthanized using CO_2_ asphyxiation and were then transcardially perfused with ice-cold PBS. Brains were carefully harvested and bisected. The left hemisphere was then drop-fixed in 4% paraformaldehyde (PFA) for 12 h at 4 °C. The right hemisphere of the brain was flash-frozen at 80 °C and stored at −80 °C. Fixed samples were placed in 30% sucrose until sunken in solution and then frozen in Tissue-Plus OCT compound (Thermo Fisher) (107). Using a cryostat (Leica), brains were sectioned at 50 μm in thickness and stored in PBS + 0.05% sodium azide at 4 °C until stained for imaging. For protein extraction, the flash-frozen brains were thawed on ice and mechanically homogenized in 500 μL tissue protein extraction reagent T-PER (Thermo Fisher, 78510) containing protease inhibitor cocktail cOmplete (Roche, 11873580001) and phosphatase inhibitor cocktail PhosSTOP (Roche, 04906845001). The brain homogenates were then spun down at 16,000 rpm for 10 min, and the supernatant was collected for soluble Aβ analysis or multiplex ELISA, while the pellets were isolated for insoluble Aβ analysis by ELISA.

### Immunofluorescence Microscopy.

As previously published (107), brain sections were blocked for 1 h at room temperature with 2% donkey serum, 1% bovine serum albumin (BSA), 0.1% triton, 0.05% tween in PBS prior to incubation with primary antibodies. The primary antibodies were then diluted in this block overnight at 4 °C. Brain sections were stained with anti-Aβ (D54D2, Cell Signaling, 1:300 dilution) to label plaques. To assess neuronal cell death, sections were stained with anti-NeuN (MAB377, Millipore Sigma, 1:500 dilution) and TUNEL (Millipore Sigma, 11684795910, according to the manufacturer’s instructions). In order to analyze microglia, sections were stained with IBA1 (ab5076, Abcam, 1:300 dilution). Microglial proliferation was assessed using Ki67-EF660 (SoIA15, Thermo Fisher, 1:100 dilution). Microglial iron accumulation was measured using Ferritin Heavy Chain (PA5-27500, Thermo Fisher, 1:500). Following primary antibody incubation, sections were then washed 3 times for 10 min at room temperature in PBS and 0.05% tween-20. After washing, the brain sections were incubated in respective donkey Alexa Fluor 488, 594, 647 anti-rabbit, -goat, -rat, -streptavidin, and -mouse (Thermo Fisher, 1:1,000 dilution) for 2 h at room temperature. Once again, brain sections were washed 3 times for 10 min at room temperature. Following washing, sections were stained with DAPI (1:1,000) for 10 min at room temperature to label nuclei, or stained with ThioflavinS (Sigma-Aldrich, 2 mg/10 mL) for 8 min followed by three 2-min washes with 50% ethanol at room temperature to label plaques (107). Brain sections were then transferred to PBS before being mounted to glass microscope slides with 50 μL ProLongGold antifade reagent (P36930, Invitrogen) and coverslips. For storage, mounted brain sections were kept at 4 °C and were imaged using LAS AF software (Leica Microsystems) on a Leica TCS SP8 confocal microscope. Images were analyzed using FIJI software or Imaris software (9.5.1).

### ELISA.

To assess Aβ composition in the brain, Aβ was measured in brain homogenates supernatants for soluble Aβ analysis, or brain homogenate pellets that underwent guanidine extraction by incubating the pellets 1:6 in 5 M guanidine HCL/50 mM tris, pH = 8.0 at room temperature for 3 h for insoluble Aβ analysis. The guanidine-extracted samples were then diluted 1:5 in PBS containing protease inhibitor cocktail cOmplete (11873580001, Roche) then centrifuged at 16,000 g for 20 min at 4 °C. The brain homogenate supernatant was diluted 1:10, and the guanidine-extraction supernatant was diluted 1:200 and quantified by Aβ 42 Mouse ELISA kit (KMB3441, Thermo Fisher). To assess cytokine levels in the brain, a multiplex ELISA was used to measure IL-1α, IL-1β, IL-4, IL-6, IL-10, IL-17, IFN-γ, and TNF-α in brain homogenate supernatants. 

### Mouse Behavior.

The MWM was completed on 4-mo-old mice and was performed as outlined in ref. 108. The first 4 d of acquisition had four 60-s trials, and the fifth day (probe) had one 60-s trial in which the hidden platform was removed. Mice were gently placed in an opaque 23 °C pool filled with white paint and a hidden platform 1 cm below water level. The pool contained 4 different visual cues with varying shapes and colors, and mice were placed in alternate places in the pool for each trial. Mice were given 60 s to find the hidden platform during each trial, however, if the mouse was unable to find the platform, they were placed on the platform for 5 s at the end of the trial. Tracking and scoring of all behavioral trials was accomplished using video tracking software (Noldus Ethovision XT).

### Aβ Oligomer Preparation.

Aβ (1-42) (641-15, California peptide) was monomerized using a previously published protocol (109), using hexafluoroisopropanol (52517, Sigma-Aldrich). Then, 5 mM monomeric Aβ samples were incubated for 24 h at 4 °C in F12 media to make a 200 μM stock of oligomeric Aβ. Samples were then incubated with CypHer5E-NHS ester (PA15401, GE Healthcare) diluted in 0.1 M sodium bicarbonate for 30 min covered and at room temperature. Following incubation, Biospin columns (7326227, Bio-Rad) were used to quench unbound dye. CypHer5E-tagged Aβ oligomers were stored at 4 °C prior to cell culture treatment or injection (107).

### In Vitro Phagocytosis.

As previously published (107), BMDMs were harvested from the hind limbs of WT and *Card9*^−/−^ mice. Collected bones were sprayed with 70% ethanol before being placed in IMDM (12440-053, Gibco) containing penicillin/streptomycin (P/S) (15140-122, Gibco). Using a 25-gauge needle, marrow was flushed through bones using 20 mL of IMDM containing P/S. Using an 18-gauge needle, the flushed bone marrow was triturated 5 times to make a single-cell suspension. Samples were spun down at 1,500 rpm for 5 min at 4 °C. Cell pellets were resuspended in bone marrow macrophages differentiation media (BMDM media) containing IMDM, 10% fetal bovine serum (FBS), 1% nonessential amino acids, 1% P/S, and 50 ng/mL M-CSF. Cells were then plated on 150 × 25-mm culture dishes (430597, Thomas Scientific). Three days after plating, 5 mL of BMDM media was added to each dish. On day 6, media was aspirated from dishes and 10 mL of PBS was added to each plate and incubated for 10 min at 4 °C. Using a scraper, BMDMs were removed from the dish and transferred to a conical tube, spun down, and resuspended in BMDM media. Two million cells per well were pipetted in 6-well plates containing glass coverslips. The next day, BMDMs were treated with 10 μM oligomeric Aβ tagged with CypHer5E for 24 h. BMDM-coated glass coverslips were fixed for 10 min at room temperature in 4% PFA. Following fixation, coverslips were washed 3 times with cold PBS. The cells were then permeabilized using 0.25% Triton X-100 diluted in PBS for 10 min at room temperature. Cells were washed 3 times with cold PBS. The coverslips containing BMDMs were then blocked in 2% donkey serum, 1% BSA, 0.1% triton, 0.05% tween in PBS for 1 h prior to incubation with primary antibodies for CD68 (MCA1957, Bio-Rad, 1:1,000 dilution) and anti-Aβ (D54D2, Cell Signaling, 1:300 dilution) diluted in blocking buffer overnight at 4 °C. Cells were then washed 3 times with PBS and stained with Alexa Fluor secondary antibodies (Thermo Fisher, 1:1,000 dilution) for 1 h at room temperature. Finally, cells were washed 3 times with PBS and stained with DAPI (1:1,000) for 10 min at room temperature before mounting the coverslips onto microscope slides to assess Aβ phagocytosis.

### In Vivo Phagocytosis.

WT and *Card9*^−/−^ mice received a 1-μL injection of CypHer5E-tagged Aβ oligomers (1 mg/mL) into the right-hemisphere cortex (at ±2 mm lateral, 0 mm anterior-posterior, and −1.5 mm ventral relative to the intersection of the coronal and sagittal suture (bregma) at a rate of 200 nL/min) using a stereotaxic frame (51730U, Stoelting) and nanoliter injector (NL2010MC2T, World Precision Instruments). Mice were euthanized according to the University of Virginia Animal Care and Use Committee using CO_2_ 48 h post injection and were transcardially perfused before processing the brains to be imaged to assess Aβ phagocytosis (107).

### Flow Cytometry.

To assess BODIPY and ROS in gated microglia, mice were euthanized using CO_2_ and transcardially perfused using 20 mL of PBS. After removing the meninges and choroid plexus, the brains were processed into a single-cell suspension as described in ref. 110. Cell pellets were then resuspended in 13 mL of 37% isotonic Percoll (17-0891-01. GE Healthcare) to remove myelin. Samples were spun down with no brake, and the myelin layer was removed. The remaining cell pellet was transferred to a 96-well V-bottom plate and washed with 1× PBS. The assessment of lipid droplet accumulation in cells was accomplished by staining the cells with BODIPY (D3861, Invitrogen, 1:2,000) diluted in PBS at 37 °C for 10 min. To label ROS, cells were stained with CellROX (C10491, Thermo Fisher, 1:500) diluted in PBS at 37 °C for 30 min. Following BODIPY and CellROX staining, cells were spun down 1,500 rpm for 5 min at 4 °C and washed with fluorescence-activated cell sorting (FACS) buffer (pH 7.4, 0.1 M PBS; 1 mM ethylenediaminetetraacetic acid (EDTA), and 1% BSA). To label microglia, cells were stained with flow antibodies for CD11b, and CD45 (eBioscience) diluted 1:200 in FACS buffer for 20 min at 4 °C. Cells were spun down and washed with FACS buffer. Prior to running samples on the cytometer, cells were resuspended in 100 μL FACS buffer containing DAPI 1:5,000. Microglia were gated as live cells (DAPI negative), single cells, and as CD11b^hi^CD45^int^ cells. BODIPY and CellROX mean fluorescence intensity was measured in these gated microglia.

### qPCR.

Trizol samples containing 20 µL of sorted microglia were thawed on ice before adding 200 μL chloroform (BP1145-1, Fisher Scientific). Samples were incubated at room temperature for 5 min and spun down at 14,000 rpm at 4 °C for 15 min. The top fraction was collected and incubated with an equal volume of isopropanol (I9516, Sigma) for 10 min at room temperature. The samples were spun down at 12,000 rpm 4 °C for 10 min, followed by 2 washes with 70% ethanol. After the last wash, ethanol was aspirated off, and the pellet was left to air dry for 15 min before adding 100 μL DNAse/RNAse free water. Sample quality was assessed using the NanoDrop 2000 Spectrophotometer (Thermo Scientific). cDNA synthesis was achieved using Sensifast cDNA Synthesis kits (BIO-65054, Bioline). Expression levels of *Klf4* (Mm00516104_m1) were determined using the Sensifast Probe No-ROX kit (BIO-86005, Bioline) and the CFX384 real-time PCR machine (1855484, BioRad) following the manufacturing protocols.

### MACS (Magnetic-Activated Cell Sorting) Isolation of Microglia for RNA-Seq.

Euthanasia of mice was performed according to the University of Virginia Animal Care and Use Committee using CO_2_ followed by transcardial perfusion using 20 mL of 1× PBS with heparin. The meninges and choroid plexus were removed from the brain before beginning the MACS protocol. Microglia were isolated using the methods described in ref. 110. In brief, brains were placed in 5 mL Hanks' balanced salt solution (HBSS) (with Mg and Ca) (14025092, Gibco) with papain 4U/mL (LS003126, Worthington) and 50 U/mL DNase I (10104159001, Sigma-Aldrich). Following 3 triturations of the samples using a 5-mL serological pipette over 45 min at 37 °C, the brain homogenates were transferred to a conical tube containing a 70-μm cell strainer and topped with 20 mL Dulbecco's modified eagle medium (DMEM)/F12 (21331020, Gibco) containing 10% FBS, 1× antibiotic-antimycotic (15240096, Thermo Fisher), and 1× GlutaMAX (35050061, Invitrogen). Strained samples were then spun down with slow brake (3 on a 0-10 scale) for 10 min at 300 G, resuspended in 160 µL MACS buffer (130-091-376, Miltenyi Biotec), and then incubated with 20 µL MACS CD11b (microglia) microbeads (130-093-634, Miltenyi Biotec) for 15 min at 4 °C. Sorting was performed using LS columns and a QuadroMACS magnet (Miltenyi, 130-042-401 and 130-091-051) according to the product instructions. The protocol efficiency was validated using flow cytometry (>90% CD11b^hi^CD45^int^) before performing RNA-seq or qPCR.

### RNA-Seq Analysis.

Microglia sorted using MACS isolation were sent to Azenta Next Generation Sequencing. Using splice-aware read aligner HISAT2, FASTQ files were aligned with the UCSC mm10 mouse genome. Quality control filtering was applied using Samtools. Next, HTSeq was used to sort reads into feature counts. DESeq2 (v1.30.0) was utilized to normalize raw counts to read depth, perform PC analysis, and carry out differential expression analysis. The Benjamini–Hochberg procedure was used to correct p-values and limit false positives arising from multiple testing. RNA-seq analyses were performed using Seq2Pathway, fgsea, tidyverse, and dplyr software packages. Heatmaps were produced using the pheatmap R package (https://github.com/raivokolde/pheatmap), lattice (http://lattice.r-forge.r-project.org/) or ggplot2 (https://ggplot2.tidyverse.org) packages. Volcano plots were produced using the EnhancedVolcano R package (https://github.com/kevinblighe/EnhancedVolcano).

### Intrahippocampal Injection Procedure.

5xFAD and *Card9*^−/−^5xFAD mice were anesthetized with a ketamine/xylazine cocktail before receiving a bilateral hippocampal injection of 2 μL of vehicle or 2 μg pustulan into the right and left hemisphere of the hippocampus (at ±2 mm lateral, −2 mm posterior, and −2 mm ventral relative to the intersection of the coronal and sagittal suture (bregma) at a rate of 200 nL/min) using a stereotaxic frame (51730U, Stoelting) and nanoliter injector (NL2010MC2T, World Precision Instruments). Seven days post injection, mice were euthanized using CO_2_ and transcardially perfused before preparing brains for immunofluorescent staining to evaluate Aβ clearance in the hippocampus. Images were analyzed using FIJI software or Imaris software (9.5.1).

### Statistics.

All statistical analyses were performed using Prism software (GraphPad). Statistical tests include Student’s *t* test (paired and unpaired), one-way ANOVA, and two-way ANOVA. *P* values less than 0.05 were deemed significant: *P <0.05, **P < 0.01, ***P < 0.001, and ****P < 0.0001. All data are represented as mean ± SEM.

## Supplementary Material

Appendix 01 (PDF)

## Data Availability

All study data are included in the article and/or *SI Appendix*. RNA-Seq data are available on the NCBI Gene Expression Omnibus (GEO) platform [GSE232636 (https://www.ncbi.nlm.nih.gov/geo/query/acc.cgi?acc=GSE232636)] (111). RNA-Seq analysis code has been deposited on Zenodo (https://zenodo.org/record/7947039#.ZGYxey-B3Up).
