# Supplementary material for: CARD9 attenuates Aβ pathology and modifies microglial responses in an Alzheimer’s disease mouse model
Source: Proc Natl Acad Sci U S A. 2023 Jun 5;120(24):e2303760120. doi: 10.1073/pnas.2303760120 (PMC10268238; doi:10.1073/pnas.2303760120)
Supplement: Supplementary file 1 — Appendix 01 (PDF) [file pnas.2303760120.sapp.pdf]

**Supporting information for**

# CARD9 attenuates Aβ pathology and modifies microglial responses in an Alzheimer's disease mouse model

Hannah Ennerfelt[1,2,3*], Coco Holliday[1], Daniel A. Shapiro[1], Kristine E. Zengeler[1,2,3], Ashley C. Bolte[1,4,5], Tyler K. Ulland[6], and John R. Lukens[1,2,3,4,5*]

[1]Center for Brain Immunology and Glia (BIG), Department of Neuroscience, University of Virginia, Charlottesville, VA 22908, USA, [2]Neuroscience Graduate Program, University of Virginia, Charlottesville, VA 22908, USA, [3]Cell and Molecular Biology Graduate Training Program, University of Virginia, Charlottesville, VA 22908, USA, [4]Department of Microbiology, Immunology and Cancer Biology, University of Virginia, Charlottesville, VA 22908, USA. [5]Medical Scientist Training Program, University of Virginia, Charlottesville, VA, 22908, USA. [6]Department of Pathology and Laboratory Medicine, University of Wisconsin, Madison, WI 53705, USA.

***Correspondence should be addressed to:**

John R. Lukens
Department of Neuroscience
Center for Brain Immunology and Glia
University of Virginia
409 Lane Road, MR4- 6154
Charlottesville VA 22908
Tel: 434-984-7782, Fax: 434-982-4380
Email: Jrl7n@virginia.edu

Hannah Ennerfelt
Department of Neuroscience
Center for Brain Immunology and Glia
University of Virginia
409 Lane Road, MR4- 6102
Charlottesville VA 22908
Tel: 434-924-7781, Fax: 434-982-4380
Email: Hee2nb@virginia.edu

**This PDF file includes:**

Figures S1 to S5

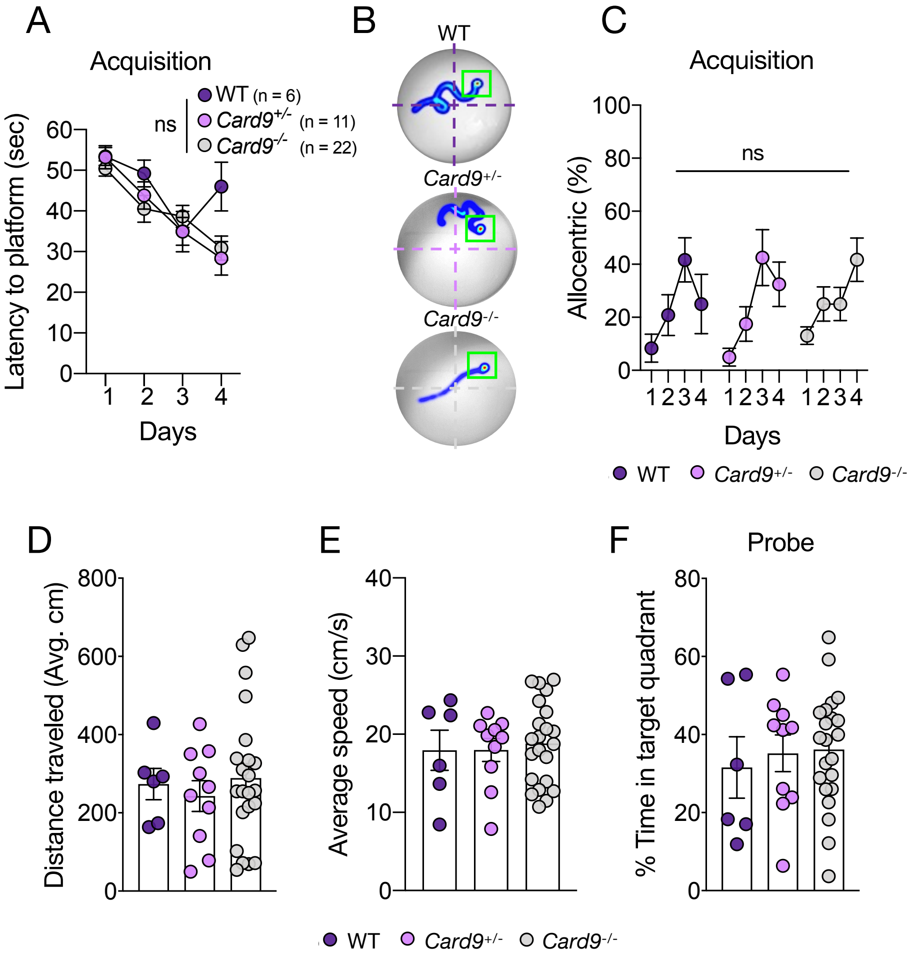


**Figure S1. Deletion of *Card9* in the absence of Aβ-mediated pathology does not impact learning or memory.** (A-F) The Morris water maze (MWM) test was used to assess spatial learning and memory in 4-month-old *Card9*$^{+/+}$ (denoted as WT), *Card9*$^{+/-}$, and *Card9*$^{-/-}$ mice. (A-E) Acquisition stage of learning in the MWM. (A) Latency to platform (acquisition). (B) Representative heatmaps of mouse trajectory on day 4 of acquisition with a green box outlining the location of the platform and (C) plotted percentage of allocentric navigation strategies during MWM acquisition. (D) Distance traveled in maze (cm) and (E) average speed of travel (cm/s) on day 4 of acquisition. (F) Percentage of time spent in the target quadrant (probe). Statistical significance between experimental groups was calculated by repeated-measures two-way ANOVA with Bonferroni's post hoc test (A, C) or one-way ANOVA with Tukey's post hoc test (D-F) from three independent experiments. ns = not significant, $^{*}P < 0.05$. Error bars represent mean ± S.E.M. (A, C-F) and each data point represents an individual mouse (D-F) or the average of experimental mice per group (A, C).

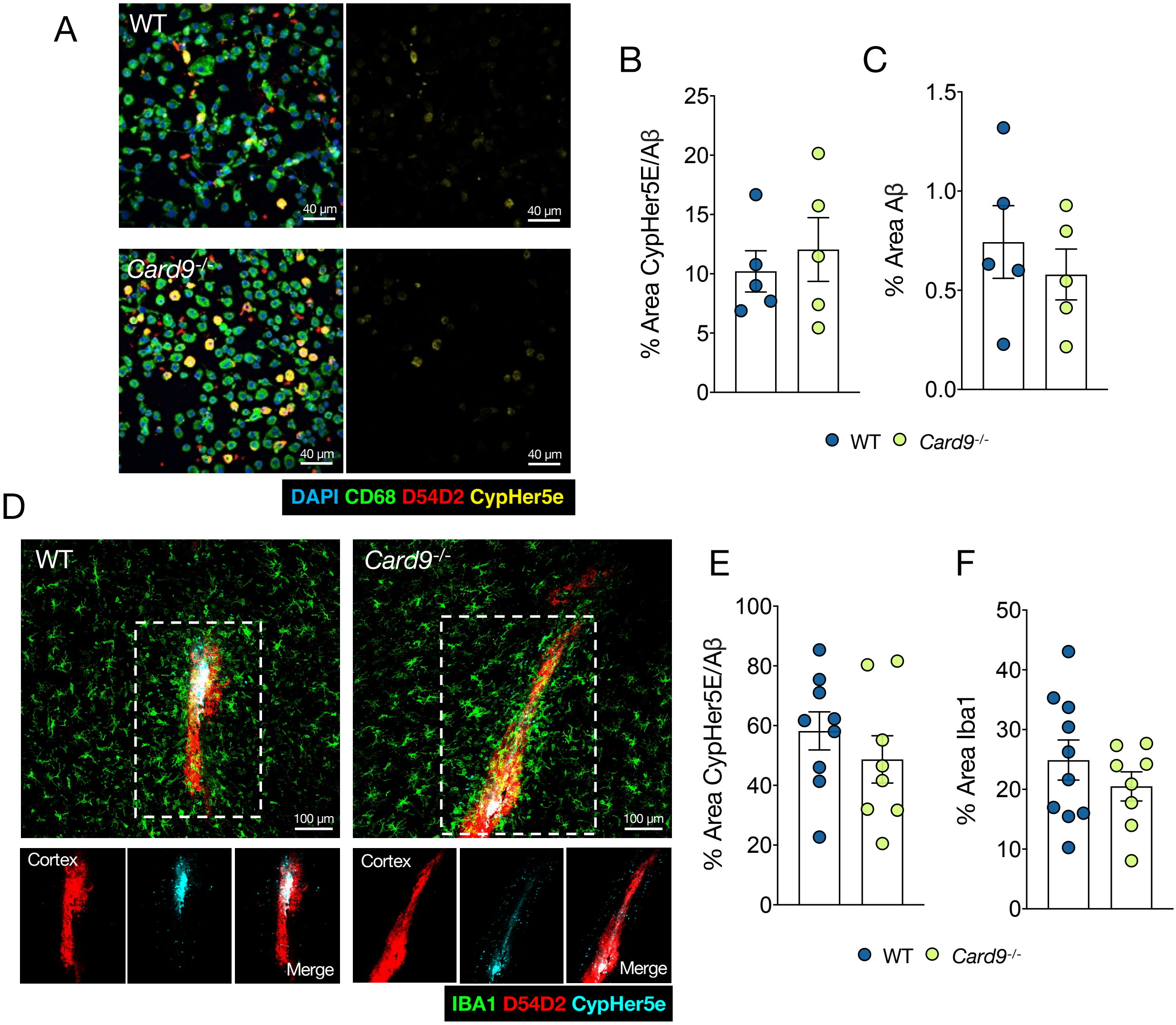


**Figure S2. CARD9-deficient mice do not display impaired Aβ clearance *in vitro* or *in vivo*.** (A-C) Bone marrow-derived macrophages (BMDMs) from C57BL/6J or *Card9*-deficient mice were stimulated with 10 µm oligomeric Aβ (D54D2, red) tagged with pH-sensitive CypHer5e dye (yellow) for 24 hours. (A) Representative images of stimulated BMDMs (CD68, green). (B) The percent area of CypHer5e dye relative to Aβ and (C) percent area of Aβ. Original magnification: 40x; scale bar = 40µm. (D-F) Stereotaxic injection of Aβ (D54D2, red) tagged with pH-sensitive CypHer5e dye (blue) in the cortex of C57BL/6J or *Card9*-deficient mice. (D) Representative images of the Aβ-CypHer5E injection site surrounded by microglia (IBA1, green) in the cortex of C57BL/6J and *Card9*-deficient mice. (E) The ratio of CypHer5e dye and residual Aβ and (F) the percent area of IBA1$^{+}$ cells at the site of injection. Consecutive sections, spaced 40 µm apart, were quantified and averaged for each mouse in a blinded fashion to assess total clearance of CypHer5E-labelled Aβ oligomers over the entire area of the injection site. Original magnification: 40x; scale bar = 100 µm. Statistical significance between experimental groups was calculated by unpaired two-tailed Student's *t*-test from two independent experiments. Error bars represent mean ± S.E.M. and each data point represents an individual mouse (B-C, E-F).

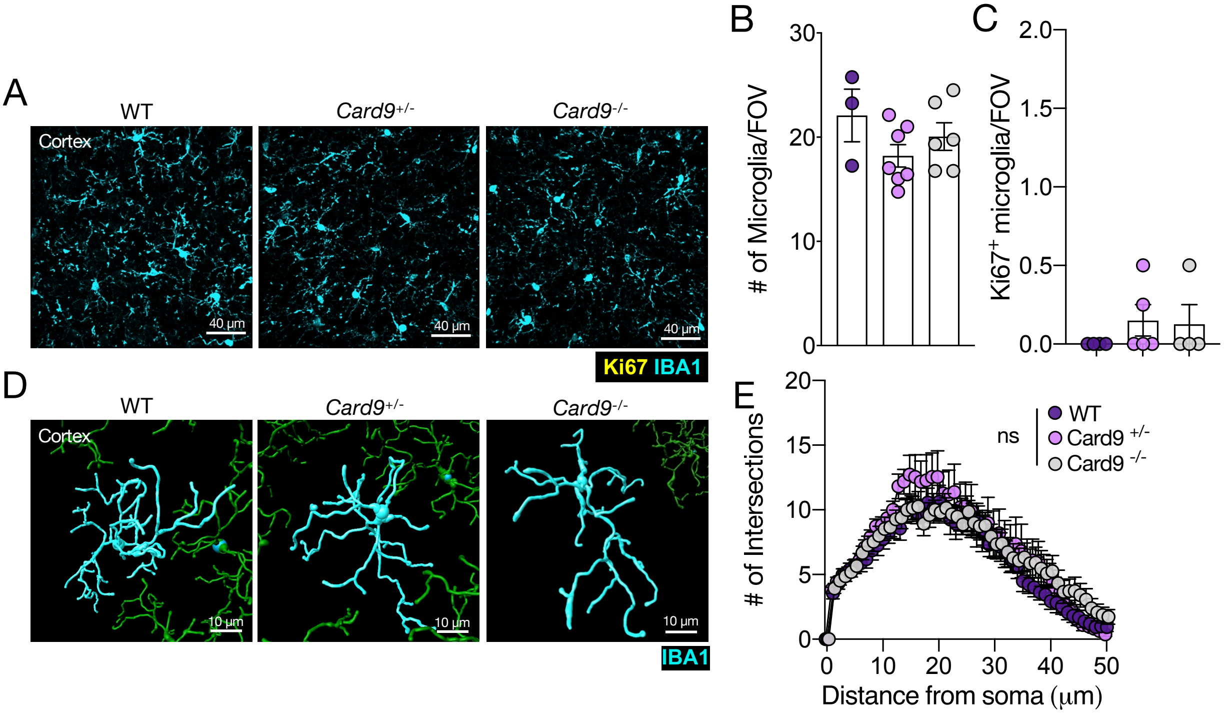


**Figure S3. *Card9* deficiency in the absence of Aβ-mediated neuropathology does not affect microglial number or morphology in adult mice.** Brains were harvested from 5-month-old *Card9*$^{+/+}$ (denoted as WT), *Card9*$^{+/-}$, and *Card9*$^{-/-}$ mice to assess microgliosis. (A-B) Representative images and quantification of microglia numbers (IBA1, cyan) in the field of view (FOV) of the frontal cortex. Original magnification: 63x; scale bar = 40 µm. (C) Quantification of microglial proliferation measured by evaluating Ki67 (yellow) colocalization with IBA1$^{+}$ (cyan) microglia in the cortex of matched sagittal sections. Data was collected from 6 fields of view (FOV) from a total of 3 matched sagittal sections. (D-E) Microglial morphology calculated by Sholl analysis from a total of 12 microglia from 3 matching brain sections per mouse (n=4 mice per group). (D) Representative microglia renderings and (E) Sholl analysis quantification. Original magnification: 63x; scale bar = 10 µm. Statistical significance between experimental groups was calculated by one-way ANOVA with Tukey's post hoc tests from two independent experiments. ns = not significant. Error bars represent mean ± S.E.M. and each data point represents an individual mouse (B-C) or represents an average of 4 mice per group (E).

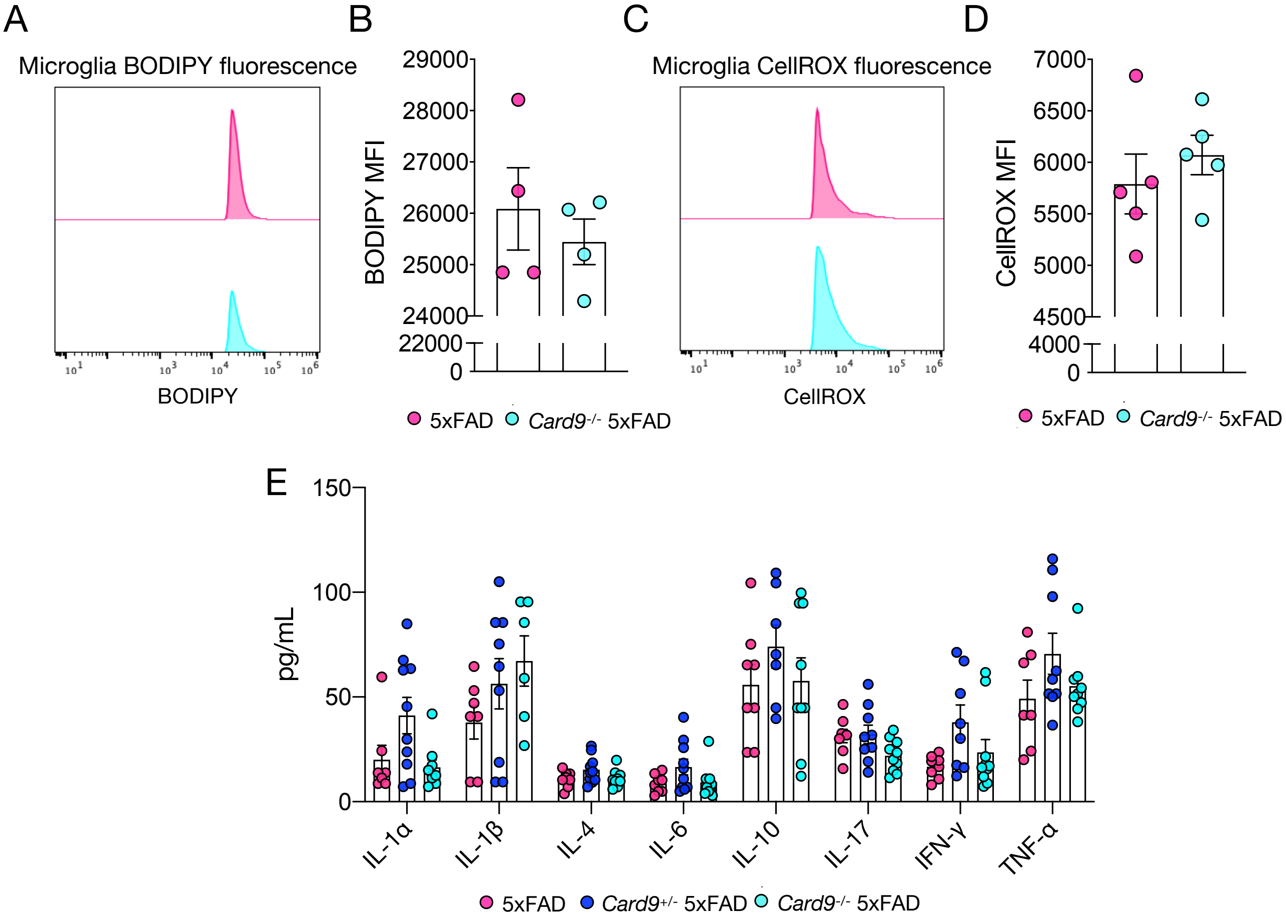


**Figure S4. *Card9* deficiency does not significantly affect microglial ROS production, lipid droplet accumulation, or expression of inflammatory cytokines associated with microglial activation in 5xFAD mice.** (A-D) $CD11b^{hi}CD45^{int}$ cells (microglia) from 5-month-old 5xFAD and *Card9*$^{-/-}$5xFAD mouse brains were probed for reactive oxygen species (ROS) production and lipid droplet accumulation by flow cytometry. (A-B) Representative flow-cytometry histograms and mean fluorescence intensity (MFI) quantification of BODIPY labeled lipid droplets in sorted $CD11b^{hi}CD45^{int}$ cells . (C-D) Representative flow-cytometry histograms and MFI quantification of CellROX labeled ROS in sorted $CD11b^{hi}CD45^{int}$ cells. (E) Brains from 5-month-old 5xFAD, *Card9*$^{+/-}$5xFAD, and *Card9*$^{-/-}$5xFAD mice were evaluated for levels of inflammation. Luminex was run to ascertain cytokine levels in the brain. Statistical significance between experimental groups was calculated by unpaired two-tailed Student's *t*-test (B, D) and one-way ANOVA with Tukey's post hoc tests (E). Error bars represent mean ± S.E.M. and each data point represents an individual mouse.

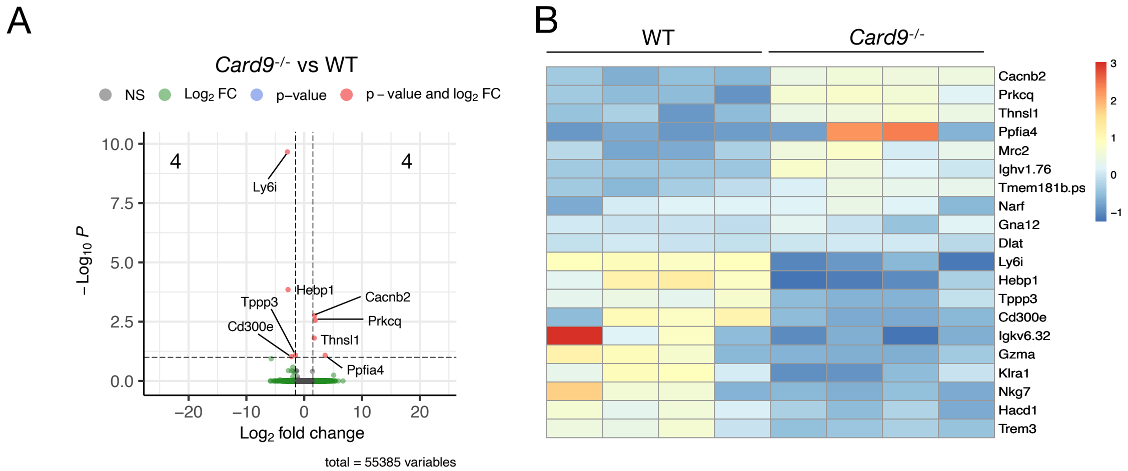


**Figure S5. *Card9* deficiency in the absence of Aβ does not appreciably affect microglial transcription.** (A-B) RNA-Seq was performed on microglia from 5-month-old *Card9*$^{+/+}$ (denoted as WT) and *Card9*$^{-/-}$ mice sorted from single-cell brain suspensions using anti-CD11b$^{+}$-coated magnetic beads and magnetic column sorting. (A) Volcano plot comparing significantly differentially expressed genes (FDR<0.1) between *Card9*$^{-/-}$ and WT microglia, where 4 genes are significantly downregulated and 4 genes are significantly upregulated. (B) Heatmap representation of the top 10 overall upregulated and downregulated genes between *Card9*$^{-/-}$ and WT microglia.
